# Processing and Bypass of a Site-Specific DNA Adduct of the Cytotoxic Platinum–Acridinylthiourea Conjugate by Polymerases Involved in DNA Repair: Biochemical and Thermodynamic Aspects

**DOI:** 10.3390/ijms221910838

**Published:** 2021-10-07

**Authors:** Monika Hreusova, Viktor Brabec, Olga Novakova

**Affiliations:** 1 Czech Academy of Sciences, Institute of Biophysics, Kralovopolska 135, CZ 61265 Brno, Czech Republic; monca.hreusova@gmail.com (M.H.); brabec@ibp.cz (V.B.); 2 Department of Biophysics, Faculty of Science, Palacky University, Slechtitelu 27, CZ 78371 Olomouc, Czech Republic

**Keywords:** cytotoxic, antitumor, platinum–acridine, lesion bypass, DNA polymerases, thermodynamic, microscale thermophoresis, translesion synthesis, drug resistance, metal–intercalator

## Abstract

DNA-dependent DNA and RNA polymerases are important modulators of biological functions such as replication, transcription, recombination, or repair. In this work performed in cell-free media, we studied the ability of selected DNA polymerases to overcome a monofunctional adduct of the cytotoxic/antitumor platinum–acridinylthiourea conjugate [PtCl(en)(L)](NO_3_)_2_ (en = ethane-1,2-diamine, L = 1-[2-(acridin-9-ylamino)ethyl]-1,3-dimethylthiourea) (ACR) in its favored 5′-CG sequence. We focused on how a single site-specific ACR adduct with intercalation potency affects the processivity and fidelity of DNA-dependent DNA polymerases involved in translesion synthesis (TLS) and repair. The ability of the G(N7) hybrid ACR adduct formed in the 5′-TCGT sequence of a 24-mer DNA template to inhibit the synthesis of a complementary DNA strand by the exonuclease-deficient Klenow fragment of DNA polymerase I (KF^exo−^) and human polymerases eta, kappa, and iota was supplemented by thermodynamic analysis of the polymerization process. Thermodynamic parameters of a simulated translesion synthesis across the ACR adduct were obtained by using microscale thermophoresis (MST). Our results show a strong inhibitory effect of an ACR adduct on enzymatic TLS: there was only small synthesis of a full-length product (less than 10%) except polymerase eta (~20%). Polymerase eta was able to most efficiently bypass the ACR hybrid adduct. Incorporation of a correct dCMP opposite the modified G residue is preferred by all the four polymerases tested. On the other hand, the frequency of misinsertions increased. The relative efficiency of misinsertions is higher than that of matched cytidine monophosphate but still lower than for the nonmodified control duplex. Thermodynamic inspection of the simulated TLS revealed a significant stabilization of successively extended primer/template duplexes containing an ACR adduct. Moreover, no significant decrease of dissociation enthalpy change behind the position of the modification can contribute to the enzymatic TLS observed with the DNA-dependent, repair-involved polymerases. This TLS could lead to a higher tolerance of cancer cells to the ACR conjugate compared to its enhanced analog, where thiourea is replaced by an amidine group: [PtCl(en)(L)](NO_3_)_2_ (complex AMD, en = ethane-1,2-diamine, L = N-[2-(acridin-9-ylamino)ethyl]-N-methylpropionamidine).

## 1. Introduction

The design of new antitumor metal-based drugs and suggestion of strategies for improving the chemotherapeutic effectiveness of the existing drugs depend on understanding the details of the molecular and biochemical mechanisms associated with the biological effects of the existing agents.

Platinum-based coordination compound cisplatin and its clinically approved and marketed analogs represent a class of successful chemotherapeutics globally used in medicine, especially as anticancer drugs [1]. Unfortunately, they are characterized by a limited effect due to the limited types of tumors they are efficient against, intrinsic resistance, severe side effects, and, in particular, acquired resistance. DNA was identified as the main pharmacological target for their action due to the formation of specific platinum complex–DNA adducts, intra-/interstrand crosslinks. Exposure of cells to genotoxic agents continually damages DNA. Therefore, DNA damage tolerance is one of the most critical factors in developing resistance and potential mutagenicity of these platinum drugs [1,2,3]. Metal–intercalator hybrid compounds represent one of the strategies for tuning biological response by changing the DNA-binding mode [4,5,6].

**Figure 1:**
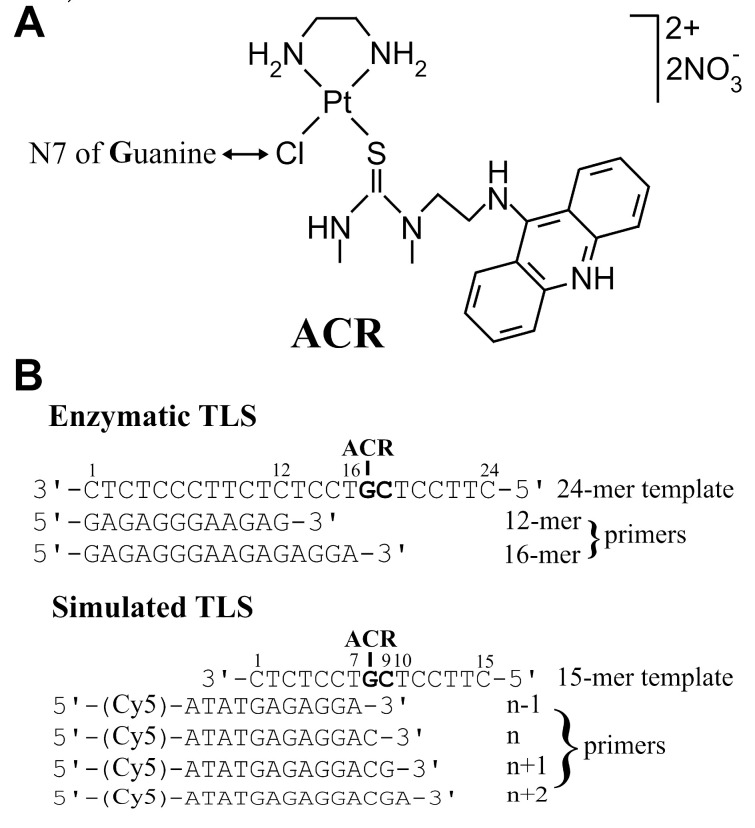
(**A**) Structure of the investigated platinum conjugate ACR, [PtCl(en)(L)](NO_3_)_2_ (en = ethane-1,2-diamine, L = 1-[2-(acridin-9-ylamino)ethyl]-1,3-dimethylthiourea), and a sketch of the platinum moiety purine coordination site. (**B**) Sequences of DNA oligonucleotides used in this study; G in the template strands represents guanine uniquely modified by the ACR conjugate in the 5′-CG sequence. Enzymatic TLS: 24-mer template (nonmodified or containing the ACR adduct) and primers for “running” or “standing” start polymerization, 12-mer or 16-mer, respectively. Simulated TLS: Set of the sequences of the 15-mer template (nonmodified or containing the ACR adduct) and n − 1, n, n + 1, n + 2 primers where n − 1 indicates the position one nucleotide before the lesion, n—position opposite the lesion, n + 1—position one nucleotide behind the lesion, and n + 2—position two nucleotides behind the lesion caused by ACR. All these primers were labelled by fluorescent dye Cy5 linked to the -ATAT- tail on the 5′ termini.

As the model system, we investigated in this study the mechanism of translesion DNA synthesis (TLS) across the cytotoxic/antitumor platinum–acridinylthiourea hybrid pharmacophore (ACR) in its favored 5′-CG sequence [7]. [PtCl(en)(L)](NO_3_)_2_ (ACR; en = ethane-1,2-diamine, L = 1-[2-(acridin-9-ylamino)ethyl]-1,3-dimethylthiourea)) (Figure 1A) is a dual metalating/intercalating DNA-binding drug that shows antiproliferative activity at micro- to nanomolar concentrations in a wide range of solid tumor cell lines [8]. The design of this conjugate was aimed at improving the efficacy of clinically established platinum drugs, i.e., cisplatin and its bifunctional derivatives. DNA perturbations produced by ACR do not appear to mimic those caused by the major cisplatin lesion, 1,2-GG intrastrand crosslink. In ~80% of adducts, ACR binds to guanine in the major groove, selectively at 5′-CG sites [7,9]. Platinum is coordinated to the N7 of guanine, and the acridine moiety intercalates into the central 5′-CG/CG base pair step on the 5′ face of the platinated guanine. The structure of the site-specifically modified dodecamer and octamer containing this adduct was already revealed and described in detail [10,11].

The mechanisms of translesion DNA synthesis (TLS) across various DNA lesions by several DNA and RNA polymerases have been studied extensively (e.g., [11,12,13,14,15,16,17,18,19]). DNA-dependent DNA and RNA polymerases are important modulators/mediators of fundamental biological functions such as replication, transcription, recombination, or repair. There is also growing evidence on the role of DNA polymerases in the origin of certain cancers; DNA polymerases can serve as new targets for cancer treatment [20,21]. Errors of DNA synthesis may occur during replication or repair because of damaged DNA; these errors are a major source of mutations since the efficiency of DNA polymerases encountering mutagenic lesions to insert a correct nucleotide may be reduced. The efficiency of DNA polymerases to insert correct nucleotides is affected not only at the site of the lesion, but also at positions several nucleotides away from the lesion, in particular in the 5′ direction [22,23,24,25,26,27]. These DNA lesions have long-range effects on polymerase activity [27,28].

TLS DNA polymerases allow cells to handle unrepaired DNA damage by promoting replication through DNA lesions that would otherwise stall the replicative polymerases [21].

The Klenow fragment deficient in exonuclease activity (KF^exo−^) was chosen for the studies reported in this article because TLS-proficient DNA polymerases of the X or Y families share some common properties, including lack of the 3′–5′ exonuclease proofreading activity. The proofreading mechanism itself may introduce effects more dependent on the lesion type [29,30,31]. Y-family DNA polymerases are notorious for bypassing DNA lesions. Human polymerase eta (polη) bypasses cisplatin–DNA adducts in a relatively efficient and error-free manner in vitro [11,32,33]. Furthermore, cell-based experiments proved that polη is involved in bypassing these adducts in vivo [34,35,36]. Polymerase kappa (polκ) is specialized for the extension step of lesion bypass. Polκ can extend mispaired primer termini and perform TLS opposite bulky DNA adducts formed by carcinogens such as benzo(a)pyrene [21,37]. Polκ is efficient and accurate in extending DNA primers after the first 3′G of the 1,2-d(GpG) cisplatin lesion [38]. Polymerase iota (polι) is distinguished from all other DNA polymerases by its exceptional fidelity. This enzyme incorporates nucleotides opposite template purines with much higher efficiency and fidelity than opposite template pyrimidines [21,39,40]. Moreover, polι is specialized to promote Hoogsteen base pairing [41], explaining polι’s ability to support replication through adducts that disrupt the Watson–Crick edge of templating purines. Polι is one of the most error-prone TLS enzymes. DNA polymerization by polκ and polι is stopped by cisplatin–DNA adducts [41,42].

DNA adducts of Pt(II)–acridine antitumor agents are relatively poor substrates for repair mechanisms [43]. ACR as the parental precursor of an improved [PtCl(en)(L)](NO_3_)_2_ (en = ethane-1,2-diamine, L = N-[2-(acridin-9-ylamino)ethyl]-N-methylpropionamidine) conjugate (AMD) was also able to inhibit human RNA polymerase II in vitro; AMD is a more potent inhibitor of RNA synthesis, which suggests that transcription inhibition may be one of the reasons for higher antiproliferative effects of AMD [43]. Despite structural differences and influence on DNA binding of these complexes, the adducts formed by both derivatives do not significantly affect the thermodynamic stability of the modified DNA [43], which plays an important role in the biological activity of and cellular response to platinum drugs [44,45,46,47,48]. The formation of monofunctional adducts increases duplex thermal stability and results in enthalpic destabilization of the 15-mer duplex, but overall does not significantly affect the free energy of duplex dissociation because of the compensatory effect of the melting (dissociation) entropies [10,43]. Energetic aspects underlying the replication and the long-range effects of the lesion on translesion synthesis across ACR have not been examined.

We investigated in this study the DNA adduct of ACR in terms of its effect on thermodynamic (TD) parameters describing the stability of DNA duplexes in the place of its origin or its immediate vicinity. We used in these experiments microscale thermophoresis (MST) which has proven to be a useful technique for obtaining TD parameters of damaged DNA [49,50,51]. The results of these thermodynamic experiments simulating TLS were compared with those of enzymatic TLS across a site-specific DNA adduct of ACR (an ability of the ACR adduct to block DNA synthesis by various DNA polymerases and/or cause a mutation) in a cell-free medium.

## 2. Results and Discussion

### 2.1. Transcription Mapping of DNA–ACR Adducts

To support and verify the relevance of the 5′-TCG sequence in the templates used in the experiments aimed at enzymatic TLS, we performed transcription mapping with the aid of SP6 and T7 RNA polymerases of the DNA–ACR adducts formed in both strands of the whole pSP73KB plasmid globally modified by ACR. We used the facts in these experiments that in vitro RNA synthesis by RNA polymerases on the DNA template containing adducts of several bifunctional Pt(II) compounds can be prematurely terminated at the level or in the proximity of the crosslinks [52,53]. Furthermore, pSP73KB DNA (part of the nucleotide sequence of this plasmid used for mapping is shown in Figure S1B) contained SP6 or T7 RNA polymerase promotors in both strands close to their 3′ ends. The experiments were carried out using DNA globally modified by the ACR conjugate at r_b_ = 0.02 or 0.01 for RNA synthesis by SP6 or T7 RNA polymerase, respectively, and by cisplatin at r_b_ = 0.01 (Figure S1A) (r_b_ is defined as the number of molecules of the platinum complex bound per nucleotide residue). RNA synthesis on the pSP73KB plasmid modified by monofunctional ACR and bifunctional cisplatin yielded fragments of defined sizes, which indicates that RNA synthesis on these templates was prematurely terminated. The major stop sites produced by the ACR platinum conjugate were mainly at guanine residues. For comparative purposes, the inhibition of RNA synthesis by DNA adducts of cisplatin is also shown and demonstrates mostly identical termination sites as those for the ACR monofunctional complex with the acridine intercalating ligand. The sequence analysis revealed that the major bands resulting from the termination of RNA synthesis by the adducts of cisplatin and ACR preferentially appear one or a half nucleotide preceding G sites and (to a considerably less extent) A sites (in AGAG, GGAG, and GAAG sequences). Summarily, the Pt(II) monofunctional-intercalating conjugate ACR exhibits base sequence selectivity similar to that of cisplatin. Nevertheless, the efficiency of the ACR adducts to terminate RNA synthesis is in general slightly reduced relative to that of cisplatin.

In vitro, RNA synthesis by RNA polymerases on this DNA template containing adducts of several bifunctional Pt(II) compounds can be prematurely terminated at the level or in the proximity of the crosslinks. On the other hand, monofunctional DNA adducts of some platinum complexes, such as [PtCl (dien)]^+^ or [PtCl(NH_3_)_3_]^+^, are unable to terminate RNA synthesis [52,53,54,55]. The monofunctional ACR conjugate formed DNA adducts that efficiently terminate RNA synthesis thanks to its bulky acridinylthiourea ligand. Differences in the chemical structure, 5′-GA/TC-binding preference of the intercalating acridine ligand [56], and the binding mode of ACR and cisplatin are manifested in some sequences. Stop sites also depend on the type of the RNA polymerase (Figure S1). ACR formed adducts mainly in the sequences 5′-TCG, 5′-CGA, and 5′-CGG. This finding is consistent with the notion that this derivative preferentially targets the 5′-cytosine–guanine step [9,57] and confirms the importance of studying TLS past the DNA–ACR adduct in the 5′-TCG sequence.

### 2.2. Enzymatic Translesion Synthesis Assays

It has been demonstrated that DNA modifications by various platinum complexes have significant effects on the processivity of a number of prokaryotic, eukaryotic, and viral DNA polymerases [11,58,59,60,61,62,63]. A lot of prokaryotic and eukaryotic DNA polymerases were blocked by site-specifically placed DNA adducts of various platinum compounds, but could also traverse through platinum adducts depending on their character and conformational alterations induced in DNA [18]. It is, therefore, of interest to examine whether TLS DNA polymerases processing a DNA substrate containing an ACR adduct in a template strand reveal differences in their efficiency and fidelity.

In this work, DNA polymerization on the DNA template containing a single site-specific adduct of the ACR conjugate was examined by DNA polymerases involved in DNA damage repair; exonuclease-deficient Klenow fragment of *E. coli* DNA polymerase I (KF^exo−^) [29] and human polymerases polη, polκ, or polι [20,21]. KF^exo−^ was selected for this study for several reasons, including because TLS-proficient DNA polymerases of the X or Y families share some common properties, including the lack of the associated 3′–5′ exonuclease proofreading activity, and the proofreading mechanism itself may introduce effects more dependent on the adduct type [31]. In eukaryotes, the Y-family DNA polymerases (polη, polκ, or polι) replicate across DNA lesions [64]. However, polι was not included in the kinetic (running- or standing-start) experiments due to its low enzymatic processivity. Therefore, polι was used only in the experiments focused on the ability of the investigated DNA polymerases to incorporate correct/incorrect nucleotides opposite unmodified/platinated guanine of the template strand under the steady-state conditions.

#### 2.2.1. Running-Start Primer Extension Experiments

**Figure 2:**
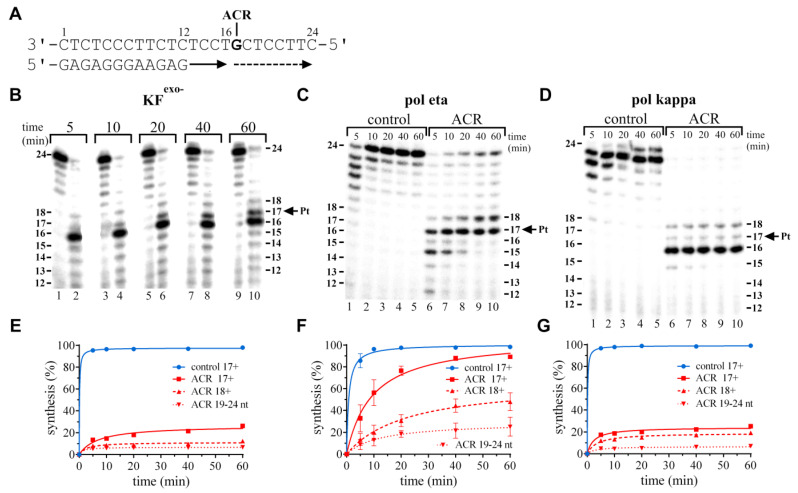
(**A**) “Running-start” translesion DNA synthesis by the exonuclease-deficient Klenow fragment of DNA polymerase I (KF^exo−^), human DNA polymerases polη and polκ on the 12-mer/24-mer primer/templates containing a single site-specific adduct of ACR in the presence of all the four dNTPs. The sequence of the primer/template duplex and the position of the platinated guanine are shown in Figure 2A. Primer extension activities of KF^exo−^, polη, and polκ are shown in Figure 2B,E, Figure 2C,F and Figure 2D,G, respectively. (**B**–**D**) Representative images of the DNA polymerase reaction products resolved on 15% polyacrylamide (PAA) gels. The experiments were conducted for various time intervals (5–60 min; shown above the gels) using the undamaged template (panel (**B**), lanes 1, 3, 5, 7, 9; panels (**C**,**D**), lanes marked as the control) and the template containing the ACR adduct (panel (**B**), lanes 2, 4, 6, 8, 10; panels (**C**,**D**), lanes marked as ACR). The pause sites and the position of the platinated guanine (intermediate lengths) are shown on the right or left side of the gels. (**E**–**G**) Densitometric evaluations of the intermediate accumulation during synthesis past the undamaged or modified template. Control 17+ and ACR 17+, translesion synthesis past and behind the position of the adduct on the undamaged (control) template (blue circles and the solid line), the template containing ACR (red squares and the solid line); ACR 18+, synthesis behind the ACR adduct (red triangles and the dashed line); ACR 19–24 nt, accumulation of “full-length” nucleotide (nt) products behind the ACR adduct (red inverted triangles and the dotted line). The data are the means (±SEM) from three different experiments.

For the translesion synthesis experiments, the 12-mer/24-mer primer/template duplexes unplatinated or containing a single site-specific adduct of the ACR conjugate were constructed (see Figure 1 and Figure 2A). The guanine in the 3′-T**G**CT-5′ sequence involved in the adduct on the template strand was located at the 17th position from the 3′ terminus (positioning the 3′ end of the primer five bases before the adduct in the template strand). DNA polymerization through the adduct of ACR by KF^exo−^ and human polymerases polη and polκ in the presence of all the four dNTPs was examined. The reaction was stopped at various time intervals, and the products were analysed using a 15%/8 M urea PAA sequencing gel.

DNA polymerization by KF^exo−^ using the gap-12-mer/24-mer primer/templates containing the adduct of ACR in the presence of all the four dNTPs proceeded rapidly up to the one nucleotide before the adduct, such that the accumulation of the 16-mer (synthesis completed one nucleotide before the adduct) corresponded to ~74% and the accumulation of the 17-mer intermediate corresponded to ~14% of all the products (Figure 2B,E). There was only small accumulation of longer DNA intermediates and “full-length” products. The products of the synthesis of a complementary DNA strand behind the platinum adduct, 18+ and 19–24 nt long, were found to correspond to ~12% and ~7% of all the products, respectively (Figure 2E). Thus, these results show that ACR forms DNA adducts, which represent a strong block of DNA polymerization by KF^exo−^.

Polymerization by human polη proceeded rapidly up to nucleotide 15 (two nucleotides before the ACR adduct) (Figure 2C,F). A considerable accumulation of intermediate products and also of “full-length” products was observed. The extent of TLS by polη was significantly higher than in the case of KF^exo−^ (~48%). Polη was able to catalyse the synthesis of “full-length” (19–24-mer) products (~25%), mostly stopped at nucleotide 17 (~41%), but there could be seen also significant accumulation of product 18, one nucleotide behind the adduct (~23%). We can summarize that the adduct of the ACR conjugate is able to effectively block DNA polymerization by polη. On the other hand, lesion bypass is 3–4 times more efficient than in the case of KF^exo−^. Chemical and/or sterical differences between enzyme active sites cause the different processing of the DNA–ACR adduct by human polη and the exonuclease-deficient Klenow fragment of DNA polymerase I in vitro.

Polymerization by human polymerase kappa using the 12-mer/24-mer primer/templates containing the adduct of ACR in the presence of all the four dNTPs proceeded rapidly up to nucleotide 16, so the 16-nucleotide intermediate product accumulated to a significant extent, ~75% (shown in Figure 2D,G). There was only a slight accumulation of longer DNA intermediates 17, 18 (~6%, ~12%, respectively), and the “full-length” (19–24 nt) product (~7%). TLS on DNA templates modified by ACR is observed, accumulation of products behind position 18 (18+) is ~19%. Accumulation of “full-length” products is as low as for KF^exo−^, i.e., ~7%. In summary, polκ replication was effectively inhibited by the ACR–DNA lesion, but still, TLS occurred. Polκ is able to extend the 12-mer primer to one nucleotide behind the modification in the template strand, then synthesizes longer DNA fragments as well. This finding is in good agreement with our results obtained with KF^exo−^ and human polη (Figure 2).

The bulky acridinylthiourea ligand of the ACR conjugate appears to be a potent inhibitor of polymerization, probably due to sterical hindrances. Intercalation between platinated G/C and the adjacent C/G base pair could help with a lesion bypass, which was possible with all the three polymerases tested, i.e., KF^exo−^, polη, and polκ; TLS mediated by polη was the most pronounced.

#### 2.2.2. Standing-Start Primer Extension Experiments

**Figure 3:**
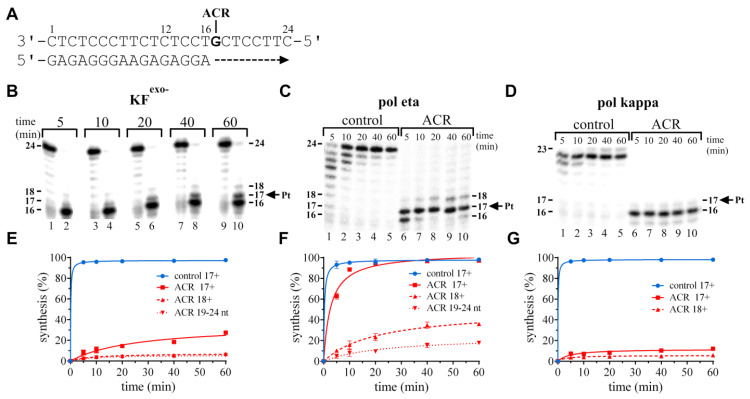
”Standing-start“ translesion DNA synthesis by the exonuclease-deficient Klenow fragment of DNA polymerase I (KF^exo−^), human DNA polymerase eta (polη), and human DNA polymerase kappa (polκ) on the 16-mer/24-mer primer/templates containing a single site-specific adduct of ACR in the presence of all the four dNTPs. The sequence of the primer/template duplex and the position of the platinated guanine is shown in panel (**A**). Primer extension activity of KF^exo−^ (**B**,**E**), polη (**C**,**F**), and polκ (**D**,**G**). (**B**–**D**) Representative images of the DNA polymerase reaction products resolved on 15% polyacrylamide (PAA) gels. The experiments were conducted for the various times (timepoints of 5–60 min are shown above the gels) using the undamaged template (panel (**B**), lanes 1, 3, 5, 7, 9; panels (**C**,**D**), control lanes) and the template containing the ACR adduct (panel (**B**), lanes 2, 4, 6, 8, 10; panels (**C**,**D**), ACR lanes). The pause sites and the position of the platinated guanine (the intermediate lengths) are shown on the right or left side of the gels. (**E**–**G**) Densitometric evaluations of the intermediate accumulation during synthesis past undamaged or modified template; 17+, translesion synthesis past and behind the position of the adduct on the undamaged (control) template (blue circles and the solid line), a template containing ACR (red squares and the solid line); 18+, synthesis behind the ACR adduct (red triangles and the dashed line); 19–24 nt, accumulation of “full-length” nucleotide (nt) products behind the ACR adduct (red inverted triangles and the dotted line). The data are the means (±SEM) from three different experiments.

For the other series of experiments, we constructed the 16-mer/24-mer primer/template duplexes unplatinated or containing adducts of the ACR conjugate (see Figure 1 and Figure 3A). The first 16 nucleotides on the 3′ terminus of the 24-mer template strand were complementary to the nucleotides of the 16-mer primer. The primer was annealed to the unplatinated or platinated template (positioning the 3′ end of the primer only one base before the adduct in the template strand: “standing-start” conditions [65]). DNA polymerization through the ACR adduct by KF^exo−^ and human polymerases in the presence of all the four dNTPs was examined in the same way as in the case of the “running-start” experiment.

Polymerization by KF^exo−^ using the 16-mer/24-mer primer/template containing the ACR adduct in the presence of all the four dNTPs proceeded mainly up to the 3′ guanine involved in the adduct. It means that one nucleotide was added, so the 17-nucleotide intermediate products accumulated to some extent (~20%) (shown in Figure 3B,E). However, there was only very slight accumulation of larger DNA intermediates (~7% of 18+ TLS). Polymerization was almost stopped at the primer, nucleotide 16 (one before the adduct) (~73%). On the other hand, it is obvious that polymerization opposite the ACR–DNA template was amplified.

Polymerization by polη on the 16-mer/24-mer substrate also proceeded mainly up to the 3′ guanine involved in the adduct, so the 17-nucleotide intermediate products accumulated to a high extent (~61%) (shown in Figure 3C,F). As can be seen from the gel, there is small accumulation of intermediate 18 (~18%), and also an accumulation of 19–24-nt products in 60 min (~18%). Polη is able to traverse over the ACR adduct and synthesize products of full length. Again, polη appears to be 3–5 times more successful in TLS than KF^exo−^. Polymerization by polκ proceeded up to the 3′ guanine involved in the adduct, and this intermediate product accumulated to some small extent (~6%) (shown in Figure 3D,G). There was no pronounced accumulation of larger intermediates, overall less than 6%. Polκ was unable to traverse effectively over the ACR adduct under the “standing-start” experimental conditions. TLS behind position 17 occurred, but the amount of products was negligible. This finding is in good agreement with polκ characteristics [21,37,38]. All the three polymerases were efficiently inhibited in the ability to bypass the ACR adduct. Nevertheless, synthesis of the 17-mer product opposite ACR modification took place. Moreover, polymerases KF^exo−^ or polη were able to synthesize 18-mer products to some extent. Formation of longer products was significantly limited; only polymerase eta accumulated ~18% of 19–24 nt long DNA fragments. Thus, polη could be responsible for some tolerance of DNA damage caused by the ACR adduct.

#### 2.2.3. Nucleotide Misinsertion Opposite the ACR Adduct by KF^exo−^ and Human Polymerases Eta, Kappa, or Iota

**Figure 4:**
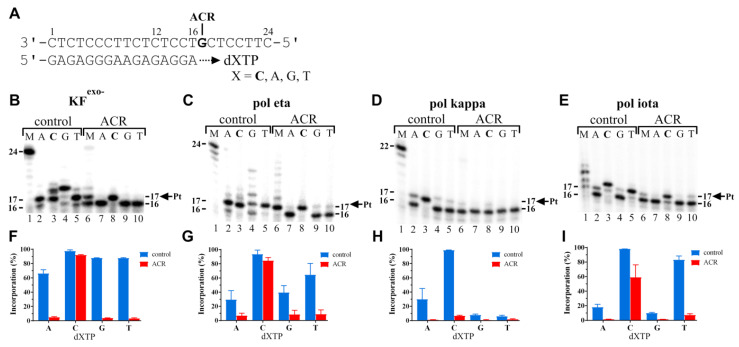
The nucleotide fidelity of the exonuclease-deficient Klenow fragment of DNA polymerase I (KF^exo−^), human DNA polymerase eta (polη), human DNA polymerase kappa (polκ), and human DNA polymerase iota (polι) on the 16-mer/24-mer primer/templates containing a single site-specific adduct of ACR in the presence of dNTPs. The sequence of the primer/template duplex and the position of the platinated guanine are shown in panel (**A**). (**B**–**E**) Representative images of the products of polymerase reactions resolved on 15% PAA gels. The capacity to elongate a 5′-^32^P-labelled 16-mer primer annealed to the unmodified 24-mer templates (control) or to the 24-mer templates containing ACR in the presence of all the four deoxyribonucleotide 5‘-triphosphates (lanes M, 25 µM each for KF^exo−^) or complementary dCTP or noncomplementary nucleotides individually (the corresponding nucleotides in these templates are marked by capital letters above the gels) at 25 °C (KF^exo−^) or 37 °C for 60 min or 120 min (polι). The pause sites (product lengths) are shown on the right or left side of the gels. The amount of synthesis was defined as the amount of radioactivity corresponding to the products of incorporation, 17–24 nucleotides long and beyond divided by the total radioactivity in the respective lane (panels (**F**–**I**)) for each sample. The data are the means (±SEM) from three different experiments.

To further analyse the impact of the ACR adduct on the enzymatic processes catalysed by KF^exo−^ or human polymerases, the nucleotide preference for incorporation opposite the 3′ platinated G involved in the adduct of ACR was examined using the 16-mer/24-mer primer/template duplexes. The polymerization reactions were performed in the presence of all the four natural deoxyribonucleotide 5′-triphosphates or a single dNTP (shown in Figure 4A) and incubated under the conditions mentioned in the legend of Figure 4 and in Chapter 4. As can be seen (Figure 4B,F), KF^exo−^ showed no strong mutational activity in the presence of the DNA template modified by ACR. Only in the case of added dCTP accumulation of intermediate 17 can be seen comparable to the control, nonmodified template.

Human polη showed some mutational activity in the presence of the DNA template modified by ACR (Figure 4C,G). This repair-associated TLS DNA polymerase was able to properly incorporate dCTP and, in addition, could replace dCTP with dTTP and, to a lesser extent, dGTP and dATP.

As can be seen (Figure 4D,H), polκ showed no strong mutational activity. Polκ could not efficiently substitute dCTP with any incorrect dNTPs. Polκ was able to incorporate correct dCTP, but only to a low extent, because of strong inhibition of polymerase kappa by monofunctional adducts of ACR. Polι (Figure 4E,I) also showed very low mutational activity in the presence of the DNA template modified by ACR. Moreover, it was used at a much higher amount (five times more than in the case of other human polymerases, polη or polκ). Moreover, the samples were incubated for 2 h instead of 1 h because the original conditions were not sufficient for polι to react. Polι could not efficiently substitute dCTP for incorrect dNTPs; however, incorporation of a small amount of dTTP was recorded (~7%). There could be seen no TLS (Figure 4E,M, lanes 1 and 6; all the four natural dNTPs were present in the samples). Polι was not able to traverse over the ACR adduct, and synthesis was blocked at nucleotide 17, opposite platinated guanine.

For a closer analysis of polymerase catalytic efficacy, we provided single-nucleotide incorporation assays, steady-state kinetic analysis. Nucleotide misinsertions (using the 16-mer/24-mer primer/template duplexes) by KF^exo−^ and human polη were quantitatively characterized by the determination of kinetic parameters of insertion of individual dNTPs. The kinetics of insertion of a single dNTP opposite the ACR adduct was determined as a function of the concentration of dNTP under steady-state conditions. For comparison, the kinetic parameters of dNMPs insertion contained in the unplatinated DNA duplex were also measured (shown in Figure S2). The steady-state apparent *K*_m_ and *V*_max_ values for the incoming dNTP incorporation opposite the template strand (with unplatinated or platinated 3′ G in the ACR adduct) were obtained from the curves fitted to the Michaelis–Menten equation as in previous papers [18,41,65,66,67]. The determined *K*_m_ and *V*_max_ parameters were used to calculate *V*_max_/*K*_m_ and, consequently, relative efficiency (*RF*) and misincorporation frequency *f* of dNMP insertions (Tables S1 and S2).

As expected, incorporation of the matched dCMP was preferred by both the selected polymerases for all the samples. Correct dCMP was incorporated with higher efficiency (*V*_max_/*K*_m_) than other incorrect nucleotides. The highest nucleotide misincorporation occurred in the control, undamaged DNA. The results are summarized in Tables S1 and S2 for KF^exo−^ and polη, respectively. In the case of KF^exo−^, all the incorrect nucleotides were incorporated opposite the ACR adduct with low relative efficiencies (*RF* = 0.01–0.07), which is in good agreement with the nucleotide misinsertion experiment (Figure 4B,F). The efficiency or misincorporation frequency was in the order dT ≥ dG >> dA for the control and dG > dT > dA for the ACR-modified template. In case of polη, the efficiency or misincorporation frequency was in the order dT > dG > dA for both the unmodified control and the ACR adduct. Higher relative efficiencies of dNMP incorporations were found for the ACR lesion processed by polη (*RF* = 0.29–0.33). Thus, polη appears to be a more “error-prone” polymerase (Figure 4C,G; Table S2).

Taken together, the adduct of ACR was processed by polymerases differently and caused low-efficiency dNTP misinsertions. On the other hand, incorporation of an incorrect nucleotide was relatively more efficient than incorporation of correct dCMP opposite modified guanine. In other words, the frequency of misinsertions was higher than that for the unmodified control duplex.

### 2.3. Simulated TLS by Microscale Thermophoresis (MST)

MST was used to explore the thermodynamics of simulated TLS across the ACR adduct at the level of the coordination site, platinated G, and in the proximity of this coordination site.

The ACR–guanine (N7) adduct in the selected 5′-TCGT sequence of the 15-mer duplex was biochemically and biophysically characterized, including thermodynamic parameters [43]. Moreover, 12-mer and 8-mer duplexes were characterized biophysically and by molecular modelling in previous studies [10,68]. It was confirmed that the monofunctional adduct of ACR enthalpically destabilized the whole duplex relatively to the unmodified control, but the Gibbs free energy was not significantly affected due to entropic compensation caused by this lesion. A significant increase of melting temperatures (*T*_m_) of the duplexes specifically modified by ACR was found: the shorter the duplex, the higher the Δ*T*_m_ (the computed difference between *T*_m_ of the ACR-modified duplex and *T*_m_ of the unmodified, control duplex). For the 15-mer, 12-mer, or 8-mer duplexes, Δ*T*_m_ = 6.4, 9.7, or 13.2 °C, respectively. Whereas the experimental conditions for the 15-mer and 12-mer duplexes were identical, the 8-mer duplex was analysed at a lower ionic strength (i.e., 150 mM NaCl vs. 10 mM NaCl). Molecular modelling of the 8-mer duplex with the ACR–guanine (N7) adduct in the 5′-CCTCGTCC sequence revealed that the modified sequence showed structural features of both B and A DNA conformation [10]. Watson–Crick hydrogen bonding stayed intact at and beyond the adduct site. Platinum was bound to the N7 position of G in the major groove, and acridine pharmacophore intercalated into the central 5′-CG/CG base pair step on the 5′ site of the platinated guanine. The chromophore’s long axis was aligned with the long axes of the adjacent base pairs, maximizing intermolecular *π*–*π* stacking interactions. The ACR adduct caused a rise (6.62 Å) and twist (15.4°) of the duplex at the central base pair step, but did not cause helical bending. No C3′-endo deoxyribose pucker and no significant roll were observed at the site of intercalation/platination [10]. The crystal structure of the ACR-modified mononucleoside deoxyguanosine indicated that combined platination of G (N7) and ideal coplanar stacking of acridine with adjacent base pairs is highly feasible [69]. In the ACR–deoxyguanosine adduct, acridine stacking stabilizes an uncommon type of GG mispairing, probably because of increased *π*-stacking interactions with a base pair compared to a single base. It was suggested that the distinct geometry observed in this model might have some relevance for the adducts formed by ACR in double-stranded DNA [69].

**Figure 5:**
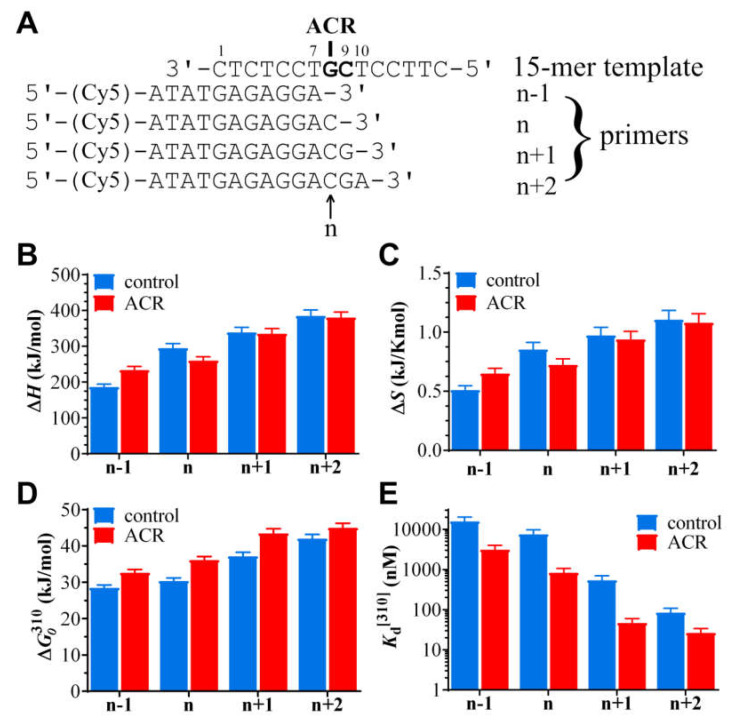
Simulated TLS: MST-derived thermodynamic parameters of dissociation of duplexes formed between the 15-mer DNA templates (unmodified, control duplex or the template platinated by ACR) and primers n − 1, n, n + 1, or n + 2. (**A**) Sequence pattern; Cy5 fluorophore is bound to the appropriate primer by the four-nucleotide linker. The GC in the template indicates the nucleobases of ACR platination (G) and intercalation of the acridine moiety (between G/C and the next C/G bp); n indicates the position of the ACR adduct in the template strand. (**B**) Diagram of enthalpy changes. (**C**) Diagram of entropy changes. (**D**) Diagram of the Gibbs free energy changes at 37 °C. (**E**) Diagram of changes of dissociation constants *K*_d_ at 37 °C.

Because MST has proven to be a good method for determining the TD parameters of DNA lesions [50,51], the appropriate template, unmodified or modified by ACR, was paired with a set of primers (from n − 1 to n + 2) (shown in Figure 1B and Figure 5A) to simulate translesion synthesis [28,48,50,51]. The primers were prolonged on the 5′ site by four nucleotides to eliminate the possible effects of Cy5 fluorophore on hybridized DNA duplexes. The experiments were performed in 10 mM phosphate buffer, pH 7, 150 mM NaCl and 0.05% Tween.

The *K*_d_ values of the primer/template hybridization (for the given appropriate temperature in the temperature range of 22–45 °C) were determined and then plotted in a Van ’t Hoff plot as ln(*K*_a_) vs. 1/*T* (Figure S3). This temperature dependence of the association constant *K*_a_ (which equals 1/*K*_d_) was used to calculate the thermodynamic parameters Δ*H*, Δ*S*, and Δ*G* (see Figure 5, Figure S3 and Table S3) as described in the experimental part and the previously published work [49,50,51].

The summarized thermodynamic data in Table S3 reveal that the *K*_d_ values of the dissociation reactions at the ambient temperature were in the nM–pM range but shifted towards significantly higher values with increasing temperature (Figure 5E). The experiments were performed with four primer sets (from n − 1 to n + 2), which differed in length. Interestingly, the hybridization affinity of all the templates and primers was raised if the templates contained the ACR adduct (Table S3).

Additionally, thermodynamic stability (expressed as Δ*G*_0_) of all the duplexes containing an ACR lesion in the template strand increased in comparison with the duplexes containing unmodified G in the control template. These results can be attributed to the intercalation of the acridine moiety of the ACR conjugate. Intercalation can be stabilized by van der Waals, hydrophobic, electrostatic, H-bond, and/or entropic interactions [6,70]. The modified n primer/template duplex was significantly enthalpically destabilized in comparison with the unmodified duplex (Figure 5B, Table S3). This finding is in good agreement with the fact that the n primer/template duplex comprises a coordination bond of the ACR conjugate. The ACR adduct caused only slight enthalpic destabilization in the duplexes n + 1 primer/template and n + 2 primer/template. It looks consistent with the data of molecular modelling showing that Watson–Crick hydrogen bonding stayed intact at and beyond the adduct site [10]. Inducing a ACR lesion caused significant enthalpic stabilization in the n − 1 primer/template duplex. It could be explained in terms of the stabilizing effect of the ACR conjugate. More precisely, the acridine moiety mediated/increased stacking interactions with an n − 1 base pair/3′-GC. This assumption is also supported by the increase of the order of the system, i.e., increase in dissociation entropy (Figure 5C, Table S3). Stacking is an important contributor to the thermodynamic stabilities of a DNA duplex [71,72]. As the observed negative change in dissociation entropy predominated for the ACR-modified duplexes, with the exception of the n − 1 primer/template duplex, hybridization of DNA strands at and behind the ACR adduct was driven entropically (Figure 5C).

There is an assumption that the insertion preference of polymerases is primarily modulated by the enthalpy term. This should be due to the suppression of enthalpy–entropy compensation in the polymerase catalytic pocket [26,73]. From this point of view, the strong block of DNA polymerization at position 17, where the ACR conjugate is coordinated to guanine(N7) in the template strand (Figure 1B, Figure 2A and Figure 3A), is in good agreement with the TD data obtained for n primer/ACR–template (Figure 1B and Figure 5A). Moreover, bypass of the ACR adduct is thermodynamically supported by an increase in enthalpy successively from n − 1 to n + 2 duplexes. In addition to that, a very low enthalpic destabilization at n + 1 primer/template and n + 2 primer/template duplexes was found (Figure 5B).

## 3. Conclusions

The ACR–DNA adduct forms a strong block for polymerases, including those involved in TLS and/or repair, and promotes an error-free mode of DNA synthesis. Polymerases from diverse families process the ACR adduct differently. As the observed negative change in dissociation entropy predominated for the ACR-modified duplexes, with the exception of the n − 1 primer/template duplex, hybridization of DNA strands at and behind the ACR adduct was driven by a change of entropy. On the other hand, no significant enthalpy destabilization of the ACR-modified duplexes n + 1 primer/template and n + 2 primer/template was found. Moreover, a successive enthalpic stabilization of the duplexes n − 1 primer/template to n + 2 primer/template occurred. That could be the reason for the TLS observed with polymerases on the ACR-modified substrates. The acridinylthiourea ligand of the ACR conjugate in a Pt–DNA monofunctional adduct appears to be an efficient inhibitor of TLS due to a sterical/bulky structure effect. Contrary, the “perfect fit” intercalation of the acridine moiety thermodynamically allows a lesion bypass.

Our results complement the picture of the action of platinum conjugates with an intercalating acridine ligand at the level of DNA damage and subsequent biological consequences; improved cytotoxic response of tumour cells to treatment with an “enhanced” AMD conjugate, where thiourea was replaced by an amidine group and where TLS behind the AMD adduct was strongly suppressed [43,51,57]. Thus, it would be possible to explain the differences between the cytotoxicity of the ACR conjugate and its AMD analogue by their different ability to overcome tumour cell resistance caused by lesion tolerance and bypass.

Our results also highlight the usefulness of MST in evaluating the impact of the dual binding mode of antitumor platinum complexes on the processing of such lesions by damaged DNA processing polymerases. Finally, the results of this work also expand the database correlating the thermodynamic characteristics of well-defined DNA damage and its mutagenic aspects.

## 4. Materials and Methods

### 4.1. Chemicals

The platinum–acridine complex ACR was synthesized and characterized according to the published procedures [57]. Stock solutions of the platinum complexes (5 × 10^−4^ M in water or NaClO_4_ (10 mM)) were stored in the dark at 4 °C. A Riboprobe^®^ System SP6/T7 for transcription mapping containing T7 and SP6 RNA polymerases was purchased from Promega (Madison, WI, USA), pSP73KB (2455 bp) plasmid was isolated according to the standard procedures. Cisplatin was obtained from Sigma-Aldrich s.r.o. (Prague, Czech Republic). Synthetic oligodeoxyribonucleotides and Cy5-labelled DNA primers were purchased from Eurofins Genomics (Ebersberg, Germany). Full-length human DNA polymerase eta (XPV protein), DNA polymerase kappa (DINB 1), and human DNA polymerase iota (RAD30B Protein) were purchased from EnzyMax, LLC (Lexington, KY, USA). The exonuclease-deficient Klenow fragment (KF^exo−^), T4 polynucleotide kinase, and dNTPs were purchased from New England Biolabs (Beverly, MA, USA). Acrylamide, bis(acrylamide), and urea were from Merck KgaA (Darmstadt, Germany). Radioactive products were from M.G.P. (Zlin, Czech Republic).

### 4.2. Transcription Mapping of DNA Platinum Adducts

Transcription of the pSP73KB plasmid DNA with SP6 or T7 RNA polymerases and electrophoretic analysis of transcripts were performed according to the protocols recommended by Promega (Promega Protocols and Applications, 43-46 (1989/90)) and were previously described in detail [52,53,54,55]. Plasmid DNA was incubated with ACR or cisplatin in 0.1 × TE buffer at 37 °C for 24 h in the dark. The number of molecules of the platinum compound coordinated per nucleotide residue (*r*_b_ values) was determined by GF AAS and spectrophotometrically. The concentration of DNA used in this assay was 7.8 × 10^−5^ M (0.5 μg/20 μL) (relative to the monomeric nucleotide content).

### 4.3. Platination of Oligonucleotides

The oligonucleotides 24-mer 5′-CTTCCTCGTCCTCTCTTCCCTCTC-3′ and 15-mer 5′-CTTCCTCGTCCTCTC-3′ were allowed to react with platinum complexes in a 1:1 molar ratio, and then the platinated oligonucleotides were purified by anion-exchange HPLC. It was verified by flameless atomic absorption spectrometry (GF AAS) and by the measurements of the optical density that the modified oligonucleotides contained one platinum atom. It was also verified using Maxam-Gilbert DMS footprinting [53,74] that one molecule of the ACR conjugate was coordinated to the N7 atom of the G in each strand of these template oligonucleotides. HPLC purification and GF AAS measurements were carried out on a Waters 600S Controller HPLC System with a MonoQ HR 5/5 column and a Varian AA240Z Zeeman atomic absorption spectrometer equipped with a graphite tube atomizer (GTA 120), respectively.

### 4.4. Translesion Synthesis Assays

The primer extension assays with all the four dNTPs were performed with the 24-mer templates containing a single monofunctional adduct of the ACR conjugate or unplatinated template, which were prepared as described above.

The 5′-^32^P-labelled primer/template DNA substrate was obtained by mixing a 12-mer 5′-GAGAGGGAAGAG-3′ or a 16-mer 5′-GAGAGGGAAGAGAGGA-3′ primer (radiolabelled at its 5′ end) with a 24-mer template 5′-CTTCCTCGTCCTCTCTTCCCTCTC-3′ at a molar ratio of 1:3 in 50 mM NaClO_4_ and hybridized for 10 min at 55 °C and for 2 h at room temperature.

All the experiments using KF^exo−^ were performed at 25 °C in 25 μL buffer containing 50 mM NaCl, 10 mM Tris HCl (pH 7.9), 10 mM MgCl_2_, 1 mM DTT, 100 μg·mL^−1^ BSA, 40 nM of the 5′-^32^P-labelled primer/template, 0.5 U (1 ng·μL^−1^) of KF^exo−^, and the four deoxynucleoside triphosphates (dNTPs) (25 μM each).

All the experiments using polη were performed at 37 °C in 25 μL buffer containing 40 mM Tris HCl (pH 8.0), 2 mM MgCl_2_, 10 mM DTT, 250 μg·mL^−1^ BSA, 60 mM KCl, 2.5% glycerol, 40 nM of the 5′-^32^P-labelled primer/template, polymerase eta (1 ng·μL^−1^), and the four deoxynucleoside triphosphates (dNTPs) (100 μM each).

All the experiments using polκ and polι were performed at 37 °C in 25 μL buffer containing 25 mM potassium phosphate (pH 7.0), 5 mM MgCl_2_, 5 mM DTT, 100 μg·mL^−1^ BSA, 10% glycerol, 40 nM of the 5′-^32^P-labelled primer/template, polκ (1 ng·μL^−1^) or polι (5 ng·μL^−1^), and the four deoxynucleoside triphosphates (dNTPs) (100 μM each).

At the appropriate time intervals (5, 10, 20, 40, and 60 min), sample aliquots (5 μL) were withdrawn, and all the enzymatic reactions were terminated by the addition of 2 μL of the stop solution containing 95% formamide, 20 mM EDTA, 0.025% bromophenol blue, and 0.025% xylene cyanol. The products were denatured by boiling at 90 °C for 1–3 min and separated by electrophoresis on a denaturing 15% polyacrylamide gel. Gels were visualized using a Typhoon FLA 7000 bioimaging analyser and analysed using the AIDA bioimage analyser software (Raytest, Straubenhardt, Germany).

### 4.5. Nucleotide Misinsertion by KF^exo−^ and Human Polymerases Eta, Kappa, Iota

Experiments were performed under the same reaction conditions as the translesion synthesis assay studies of individual polymerases in the steady state (60 min or 120 min for polι) in the presence of all the four deoxyribonucleotide 5′-triphosphates or selected dNTPs, complementary dCTP, or noncomplementary dATP, dGTP, and dTTP (100 μM each). The reactions were terminated as described above.

### 4.6. Steady-State Kinetic Analysis for dNTP Incorporations by KF^exo−^ and Polη

Steady-state kinetic analysis for dNTP incorporation opposite the unplatinated or platinated G (in the template, 3′ oligonucleotide with the monofunctional adduct of ACR) catalysed by KF^exo−^ and human polη was performed as described previously [41,65,66,67]. The same amount of polη and KF^exo−^, under the same reaction conditions as in the nucleotide fidelity experiments mentioned above, was incubated with a hybridized 16-mer primer/template in the presence of individual dNTPs (increasing concentration of 0.125–1000 µM of the examined dNTP) for 10 min. The reactions were terminated as in the previous experiments. Gel band intensities of the substrates and products were visualized and quantified. The percentage of the 16-mer primer extended (the sum of the intensities of the bands corresponding to all the 17 and/or extended 17+ products [41,65,66,67]) was plotted as a function of product concentration, and the data were fitted by nonlinear regression using the GraphPad software to the Michaelis–Menten equation describing a hyperbola, v = (*V*_max_ × [dNTP]/*K*_m_ + [dNTP]).

Apparent *K*_m_ and *V*_max_ steady-state parameters were obtained from the best fit and were used to calculate the relative efficiency (*RF*) of the dNTP insertion opposite the template G: (*V*_max_/*K*_m_)_platinated template_/(*V*_max_/*K*_m_)_unplatinated template_. The relative dNMP mutagenicity for the oligonucleotides with the ACR adduct and the control unplatinated oligonucleotide was expressed as a ratio of the respective misincorporation frequency *f* = (*V*_max_/*K*_m_)_incorrect nucleotide_/(*V*_max_/*K*_m_)_correct nucleotide_.

### 4.7. MST-Derived Thermodynamic Parameters

Microscale thermophoresis (MST) is a suitable technique for obtaining thermodynamic (TD) parameters of modified oligonucleotides [49,50,51]. We measured the DNA hybridization equilibrium binding constant *K*_a_ of a 15-mer oligonucleotide and a Cy5-labelled complementary primer over a range of temperatures (22–45 °C) using a Monolith NT115 Pico instrument. DNA hybridization can be readily detected by MST due to the different thermophoretic signals of ssDNA and dsDNA.

Serial dilution of platinated 15-mer 5′-CTTCCTCGTCCTCTC-3′ and unplatinated templates (1 × 10^−5^ M–3 × 10^−11^ M) were paired with a set of 5’-Cy5-labelled primers (from n − 1 to n + 2) 5′-ATATGAGAGGA-3′, 5′-ATATGAGAGGAC-3′, 5′-ATATGAGAGGACG-3′, 5′-ATATGAGAGGACGA-3′ (2–8 nM) in the volume ratio 1:1 in 10 mM phosphate buffer (pH = 7.0), 150 mM NaCl and 0.05% Tween.

All the reaction solutions were filled into the standard glass capillary tubes and immediately measured with the MST instrument. Data analyses and curve fitting were carried out using the Nanotemper Analysis software. All the experimental parameters used by the MST instrument were fixed with the LED power of 20–100% and the medium laser power of 40%. The *K*_d_ values for each temperature were determined and plotted in a Van ’t Hoff plot as ln(*K*_a_) vs. 1/*T*. The temperature dependence of the association constant *K*_a_ (which equals 1/*K*_d_) was used to deduce the thermodynamic parameters Δ*H* and Δ*S* by linear extrapolation of the data in the Van ’t Hoff plot. Δ*G* was calculated by using equation Δ*G* = Δ*H* − *T*Δ*S*. Thermodynamic parameters of the platinated oligonucleotide were compared with the unplatinated control.

## Data Availability

Data is contained within the article or Supplementary Materials.

## Supplementary Materials

The following are available online at https://www.mdpi.com/article/10.3390/ijms221910838/s1.

## Abbreviations

HPLC	High-performance liquid chromatography
GF AA	Graphite furnace atomic absorption spectrometry
EAS	Electronic absorption spectrophotometry
TLS	Translesion DNA synthesis
KF^exo−^	Klenow fragment of DNA polymerase I (the exonuclease deficient)
PAA	Polyacrylamide
MST	Microscale thermophoresis
DTT	Dithiothreitol
BSA	Bovine serum albumin
dNTP	2′-deoxyribonucleotide-5′-triphosphate
dNMP	2′-deoxyribonucleotide-5′-monophosphate
ACR	[PtCl(en)(L)](NO_3_)_2_ (en = ethane-1,2-diamine, L = 1-[2-(acridin-9-ylamino)ethyl]-1,3-dimethylthiourea) complex
AMD	[PtCl(en)(L)](NO_3_)_2_ (en = ethane-1,2-diamine, L = *N*-[2-(acridin-9-ylamino)ethyl]-*N*-methylpropionamidine) complex
cisplatin	*cis*-[Pt(NH_3_)_2_Cl_2_] (*cis*-diamminedichloridoplatinum(II))
Cy5	Cyanine dye (1,1′-bis(3-hydroxypro-pyl)-3,3,3′,3′-tetramethylindodicarbocyanine)
pol	Polymerase
nt	Nucleotide
bp	Base pair

## References

[1] Johnstone T.C., Suntharalingam K., Lippard S.J. (2016). The Next Generation of Platinum Drugs: Targeted Pt (II) Agents, Nanoparticle Delivery, and Pt (IV) Prodrugs. Chem. Rev..

[2] Kelland L. (2007). The resurgence of platinum-based cancer chemotherapy. Nat. Rev. Cancer.

[3] Todd R.C., Lippard S.J. (2009). Inhibition of transcription by platinum antitumor compounds. Metallomics.

[4] Brabec V., Hrabina O., Kasparkova J. (2017). Cytotoxic platinum coordination compounds. DNA binding agents. Coord. Chem. Rev..

[5] Baruah H., Barry C.G., Bierbach U. (2004). Platinum-intercalator conjugates: From DNA-targeted cisplatin derivatives to adenine binding complexes as potential modulators of gene regulation. Curr. Top. Med. Chem..

[6] Zeglis B.M., Pierre V.C., Barton J.K. (2007). Metallo-intercalators and metallo-insertors. Chem. Commun..

[7] Barry C.G., Baruah A.H., Bierbach U. (2003). Unprecedented Monofunctional Metalation of Adenine Nucleobase in Guanine- and Thymine-Containing Dinucleotide Sequences by a Cytotoxic Platinum−Acridine Hybrid Agent. J. Am. Chem. Soc..

[8] Guddneppanavar R., Bierbach U. (2007). Adenine-N3 in the DNA minor groove—An emerging target for platinum containing anticancer pharmacophores. Anti-Cancer Agents Med. Chem..

[9] Budiman M.E., Alexander R.W., Bierbach U. (2004). Unique base-step recognition by a platinum-acridinylthiourea conjugate leads to a DNA damage profile complementary to that of the anticancer drug cisplatin. Biochemistry.

[10] Baruah H., Wright M.W., Bierbach U. (2005). Solution structural study of a DNA duplex containing the guanine-N7 adduct formed by a cytotoxic platinum-acridine hybrid agent. Biochemistry.

[11] Bassett E., Vaisman A., Havener J.M., Masutani C., Hanaoka F., Chaney S.G. (2003). Efficiency of extension of mismatched primer termini across from cisplatin and oxaliplatin adducts by human DNA polymerases beta and eta in vitro. Biochemistry.

[12] Arana M.E., Song L., Le Gac N.T., Parris D.S., Villani G., Boehmer P.E.B. (2004). On the role of proofreading exonuclease in bypass of a 1,2 d(GpG) cisplatin adduct by the herpes simplex virus-1 DNA polymerase. DNA Repair.

[13] Wickramaratne S., Boldry E.J., Buehler C., Wang Y.-C., Distefano M.D., Tretyakova N.Y. (2015). Error-prone translesion synthesis past DNA-peptide cross-links conjugated to the major groove of DNA via C5 of thymidine. J. Biol. Chem..

[14] O’Flaherty D.K., Guengerich F.P., Egli M., Wilds C.J. (2015). Backbone flexibility influences nucleotide incorporation by human translesion DNA polymerase η opposite intrastrand cross-linked DNA. Biochemistry.

[15] Villani G., Hubscher U., Gironis N., Parkkinen S., Pospiech H., Shevelev I., di Cicco G., Markkanen E., Syvaoja J.E., Tanguy Le Gac N. (2011). In vitro gap-directed translesion DNA synthesis of an abasic site involving human DNA polymerases epsilon, lambda, and beta. J. Biol. Chem..

[16] Ho T.V., Guainazzi A., Derkunt S.B., Enoiu M., Schärer O.D. (2011). Structure-dependent bypass of DNA interstrand crosslinks by translesion synthesis polymerases. Nucleic Acids Res..

[17] Beard W.A., Wilson S.H. (2003). Structural insights into the origins of DNA polymerase fidelity. Structure.

[18] Kasparkova J., Suchankova T., Halamikova A., Zerzankova L., Vrana O., Margiotta N., Natile G., Brabec V. (2010). Cytotoxicity, cellular uptake, glutathione and DNA interactions of an antitumor large-ring Pt^II^ chelate complex incorporating the cis-1,4-diaminocyclohexane carrier ligand. Biochem. Pharmacol..

[19] Novakova O., Farrell N.P., Brabec V. (2018). Translesion DNA synthesis across double-base lesions derived from cross-links of an antitumor trinuclear platinum compound: Primer extension, conformational and thermodynamic studies. Metallomics.

[20] Hubscher U., Maga G., Spadari S. (2002). Eukaryotic DNA polymerases. Annu. Rev. Biochem..

[21] Jain R., Aggarwal A.K., Rechkoblit O. (2018). Eukaryotic DNA polymerases. Curr. Opin. Struct. Biol..

[22] Ng L., Weiss S.J., Fisher P.A. (1989). Recognition and binding of template-primers containing defined abasic sites by Drosophila DNA polymerase alpha holoenzyme. J. Biol. Chem..

[23] Weiss S.J., Fisher P.A. (1992). Interaction of Drosophila DNA polymerase alpha holoenzyme with synthetic template-primers containing mismatched primer bases or propanodeoxyguanosine adducts at various positions in template and primer regions. J. Biol. Chem..

[24] Lindsley J.E., Fuchs R.P.P. (1994). Use of single-turnover kinetics to study bulky adduct bypass by T7 DNA polymerase. Biochemistry.

[25] Miller H., Grollman A.P. (1997). Kinetics of DNA polymerase I (Klenow fragment exo-) activity on damaged DNA templates: Effect of proximal and distal template damage on DNA synthesis. Biochemistry.

[26] Minetti C., Remeta D.P., Miller H., Gelfand C.A., Plum G.E., Grollman A.P., Breslauer K.J. (2003). The thermodynamics of template-directed DNA synthesis: Base insertion and extension enthalpies. Proc. Natl. Acad. Sci. USA.

[27] Liang F., Cho B.P. (2007). Probing the thermodynamics of aminofluorene-induced translesion DNA synthesis by differential scanning calorimetry. J. Am. Chem. Soc..

[28] Florian J., Brabec V. (2012). Thermodynamics of translesion synthesis across a major DNA adduct of antitumor oxaliplatin: Differential scanning calorimetric study. Chem. Eur. J..

[29] Turner R.M., Grindley N.D.F., Joyce C.M. (2003). Interaction of DNA polymerase I (Klenow fragment) with the single-stranded template beyond the site of synthesis. Biochemistry.

[30] Patel P.H., Suzuki M., Adman E., Shinkai A., Loeb L.A. (2001). Prokaryotic DNA polymerase I: Evolution, structure, and “base flipping” mechanism for nucleotide selection. J. Mol. Biol..

[31] Villani G., Le Gac N.T., Wasungu L., Burnouf D., Fuchs R.P., Boehmer P.E. (2002). Effect of manganese on in vitro replication of damaged DNA catalyzed by the herpes simplex virus type-1 DNA polymerase. Nucleic Acids. Res..

[32] Vaisman A., Masutani C., Hanaoka F., Chaney S.G. (2000). Efficient translesion replication past oxaliplatin and cisplatin GpG adducts by human DNA polymerase η. Biochemistry.

[33] Bassett E., Vaisman A., Tropea K.A., McCall C.M., Masutani C., Hanaoka F., Chaney S.G. (2002). Frameshifts and deletions during in vitro translesion synthesis past Pt-DNA adducts by DNA polymerases beta and eta. DNA Repair.

[34] Yamada K., Takezawa J., Ezaki O. (2003). Translesion replication in cisplatin-treated xeroderma pigmentosum variant cells is also caffeine-sensitive: Features of the error-prone DNA polymerase(s) involved in UV-mutagenesis. DNA Repair.

[35] Bassett E., King N.M., Bryant M.F., Hector S., Pendyala L., Chaney S.G., Cordeiro-Stone M. (2004). The role of DNA polymerase eta in translesion synthesis past platinum-DNA adducts in human fibroblasts. Cancer Res..

[36] Albertella M.R., Green C.M., Lehmann A.R., O’Connor M.J. (2005). A role for polymerase eta in the cellular tolerance to cisplatin-induced damage. Cancer Res..

[37] Vaisman A., Woodgate R. (2017). Translesion DNA polymerases in eukaryotes: What makes them tick?. Crit. Rev. Biochem. Mol. Biol..

[38] Jha V., Ling H. (2018). Structural basis for human DNA polymerase kappa to bypass cisplatin intrastrand cross-link (Pt-GG) lesion as an efficient and accurate extender. J. Mol. Biol..

[39] Tissier A., McDonald J.P., Frank E.G., Woodgate R. (2000). Polι, a remarkably error-prone human DNA polymerase. Genes Dev..

[40] McDonald J.P., Tissier A., Frank E.G., Iwai S., Hanaoka F., Woodgate R. (2001). DNA polymerase iota and related Rad30-like enzymes. Philos. Trans. R. Soc. Lond. B Biol. Sci..

[41] McIntyre J. (2020). Polymerase iota-an odd sibling among Y family polymerases. DNA Repair.

[42] Brown J.A., Newmister S.A., Fiala K.A., Suo Z. (2008). Mechanism of double-base lesion bypass catalyzed by a Y-family DNA polymerase. Nucleic Acids Res..

[43] Kostrhunova H., Malina J., Pickard A.J., Stepankova J., Vojtiskova M., Kasparkova J., Muchova T., Rohlfing M.L., Bierbach U., Brabec V. (2011). Replacement of a thiourea with an amidine group in a monofunctional platinum-acridine antitumor agent. Effect on DNA interactions, DNA adduct recognition and repair. Mol. Pharm..

[44] Pilch D.S., Dunham S.U., Jamieson E.R., Lippard S.J., Breslauer K.J. (2000). DNA sequence context modulates the impact of a cisplatin 1,2-d(GpG) intrastrand cross-link and the conformational and thermodynamic properties of duplex DNA. J. Mol. Biol..

[45] Hofr C., Farrell N., Brabec V. (2001). Thermodynamic properties of duplex DNA containing a site-specific d(GpG) intrastrand cross-link formed by an antitumor dinuclear platinum complex. Nucleic Acids Res..

[46] Malina J., Novakova O., Vojtiskova M., Natile G., Brabec V. (2007). Conformation of DNA GG intrastrand cross-link of antitumor oxaliplatin and its enantiomeric analog. Biophys. J..

[47] Bursova V., Kasparkova J., Hofr C., Brabec V. (2005). Effects of monofunctional adducts of platinum (II) complexes on thermodynamic stability and energetics of DNA duplexes. Biophys. J..

[48] Malina J., Novakova O., Natile G., Brabec V. (2012). The thermodynamics of translesion DNA synthesis past major adducts of enantiomeric analogues of antitumor cisplatin. Chem. Asian J..

[49] Jerabek-Willemsen M., André T., Wanner R., Roth H.M., Duhr S., Baaske P., Breitsprecher D. (2014). MicroScale Thermophoresis: Interaction analysis and beyond. J. Mol. Struct..

[50] Hrabina O., Brabec V., Novakova O. (2019). Translesion DNA synthesis across lesions induced by oxidative products of pyrimidines. An insight into the mechanism by microscale thermophoresis. Int. J. Mol. Sci..

[51] Hreusova M., Novakova O., Brabec V. (2020). Thermodynamic insights by microscale thermophoresis into translesion DNA synthesis catalyzed by DNA polymerases across a lesion of antitumor platinum–acridine complex. Int. J. Mol. Sci..

[52] Lemaire M.A., Schwartz A., Rahmouni A.R., Leng M. (1991). Interstrand cross-links are preferentially formed at the d(GC) sites in the reaction between *cis*-diamminedichloroplatinum (II) and DNA. Proc. Natl. Acad. Sci. USA.

[53] Brabec V., Leng M. (1993). DNA interstrand cross-links of trans-diamminedichloroplatinum (II) are preferentially formed between guanine and complementary cytosine residues. Proc. Natl. Acad. Sci. USA.

[54] Novakova O., Malina J., Kasparkova J., Halamikova A., Bernard V., Intini F., Natile G., Brabec V. (2009). Energetics, conformation, and recognition of DNA duplexes modified by methylated analogues of [PtCl(dien)]^+^. Chem. Eur. J..

[55] Betanzos-Lara S., Salassa L., Habtemariam A., Novakova O., Pizarro A.M., Clarkson G.J., Liskova B., Brabec V., Sadler P.J. (2012). Photoactivatable organometallic pyridyl ruthenium (II) arene complexes. Organometallics.

[56] Baruah H., Bierbach U. (2003). Unusual intercalation of acridin-9-ylthiourea into the 5′-GA/TC DNA base step from the minor groove: Implications for the covalent DNA adduct profile of a novel platinum-intercalator conjugate. Nucleic Acids. Res..

[57] Ma Z., Choudhury J.R., Wright M.W., Day C.S., Saluta G., Kucera G.L., Bierbach U. (2008). A non-cross-linking platinum-acridine agent with potent activity in non-small-cell lung cancer. J. Med. Chem..

[58] Kasparkova J., Novakova O., Marini V., Najajreh Y., Gibson D., Perez J.-M., Brabec V. (2003). Activation of trans geometry in bifunctional mononuclear platinum complexes by a piperidine ligand: Mechanistic studies on antitumor action. J. Biol. Chem..

[59] Comess K.M., Burstyn J.N., Essigmann J.M., Lippard S.J. (1992). Replication inhibition and translesion synthesis on templates containing site-specifically placed *cis*-diamminedichloroplatinum (II) DNA adducts. Biochemistry.

[60] Suo Z., Johnson K. (1998). DNA secondary structure effects on DNA synthesis catalyzed by HIV-1 reverse transcriptase. J. Biol. Chem..

[61] Vaisman A., Warren M.W., Chaney S.G. (2001). The effect of DNA structure on the catalytic efficiency and fidelity of human DNA polymerase beta on templates with platinum-DNA adducts. J. Biol. Chem..

[62] Moriarity B., Novakova O., Farrell N., Brabec V., Kasparkova J. (2007). 1,2-GG intrastrand cross-link of antitumor dinuclear bifunctional platinum compound with spermidine linker inhibits DNA polymerization more effectively than the cross-link of conventional cisplatin. Arch. Biochem. Biophys..

[63] Alt A., Lammens K., Chiocchini C., Lammens A., Pieck J.C., Kuch D., Hopfner K.P., Carell T. (2007). Bypass of DNA lesions generated during anticancer treatment with cisplatin by DNA polymerase. Science.

[64] Prakash S., Johnson R.E., Prakash L. (2005). Eukaryotic translesion synthesis DNA polymerases: Specificity of structure and function. Annu. Rev. Biochem..

[65] Creighton S., Bloom L.B., Goodman M.F. (1995). Gel fidelity assay measuring nucleotide misinsertion, exonucleolytic proofreading, and lesion bypass efficiencies. Methods Enzymol..

[66] Mendelman L., Petruska J., Goodman M. (1990). Base mispair extension kinetics. Comparison of DNA polymerase and reverse transcriptase. J. Biol. Chem..

[67] Goodman M.F., Creighton S., Bloom L.B., Petruska J., Kunkel T.A. (1993). Biochemical basis of DNA replication fidelity. Crit. Rev. Biochem. Mol. Biol..

[68] Baruah H., Bierbach U. (2004). Biophysical characterization and molecular modeling of the coordinative-intercalative DNA monoadduct of a platinum-acridinylthiourea agent in a site-specifically modified dodecamer. J. Biol. Inorg. Chem..

[69] Baruah H., Day C.S., Wright M.W., Bierbach U. (2004). Metal-intercalator-mediated self-association and one-dimensional aggregation in the structure of the excised major DNA adduct of a platinum-acridine agent. J. Am. Chem. Soc..

[70] Gill M.R., Thomas J.A. (2012). Ruthenium (II) polypyridyl complexes and DNA-from structural probes to cellular imaging and therapeutics. Chem. Soc. Rev..

[71] Bommarito S., Peyret N., SantaLucia J. (2000). Thermodynamic parameters for DNA sequences with dangling ends. Nucleic Acids. Res..

[72] Yakovchuk P., Protozanova E., Frank-Kamenetskii M.D. (2006). Base-stacking and base-pairing contributions into thermal stability of the DNA double helix. Nucleic Acids. Res..

[73] Petruska J., Goodman M.F., Boosalis M.S., Sowers L.C., Cheong C., Tinoco I. (1988). Comparison between DNA melting thermodynamics and DNA polymerase fidelity. Proc. Natl. Acad. Sci. USA.

[74] Kasparkova J., Mellish K.J., Qu Y., Brabec V., Farrell N. (1996). Site-specific d(GpG) intrastrand cross-links formed by dinuclear platinum complexes. Bending and NMR studies. Biochemistry.

