# Supplementary material for: Processing and Bypass of a Site-Specific DNA Adduct of the Cytotoxic Platinum–Acridinylthiourea Conjugate by Polymerases Involved in DNA Repair: Biochemical and Thermodynamic Aspects
Source: Int J Mol Sci. 2021 Oct 7;22(19):10838. doi: 10.3390/ijms221910838 (PMC8509567; doi:10.3390/ijms221910838)
Supplement: Supplementary file 1 [file ijms-22-10838-s001.zip › ijms-1328993-supplementary.pdf]

Supplementary material to:

Processing and Bypass of a Site-Specific DNA Adduct of the Cytotoxic Platinum–Acridinylthiourea Conjugate by Polymerases Involved in DNA Repair: Biochemical and Thermodynamic Aspects

Monika Hreusova [1], Viktor Brabec [1,2] and Olga Novakova [1,*]

[1] Czech Academy of Sciences, Institute of Biophysics, Kralovopolska 135, CZ- 61265 Brno, Czech Republic; olga@ibp

[2] Department of Biophysics, Faculty of Science, Palacky University, Slechtitelu 27, CZ 78371 Olomouc, Czech Republic; brabec@ibp.cz

* Correspondence: olga@ibp.cz; Tel.: +420-541-517-135

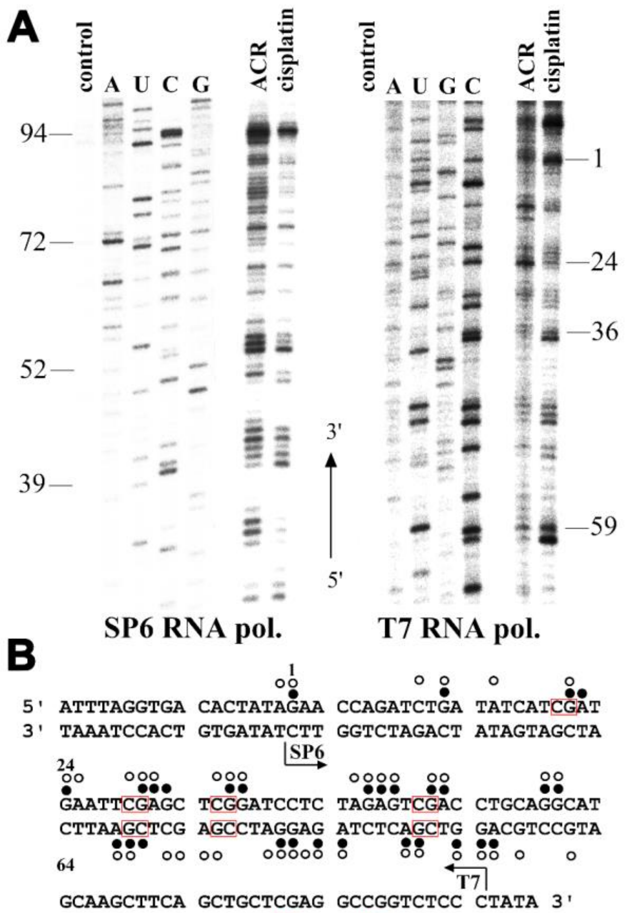

Figure S1. Inhibition of RNA synthesis by SP6 and T7 RNA polymerases on the whole pSP73KB plasmid modified by ACR, monofunctional platinum conjugate, and cisplatin. A. Autoradiograms of 8 % polyacrylamide/8 M urea sequencing gels showing inhibition of RNA synthesis by SP6 (left) or T7 RNA polymerases (right) on the plasmid DNA containing adducts of platinum complexes. Lanes: control, unmodified template; A, U, C, and G, chain terminated marker RNAs; ACR and cisplatin, the template modified by Pt(II)-ACR at r_b = 0.016 (SP6 RNA pol.) or r_b = 0.009 (T7 RNA pol.), or cisplatin at r_b = 0.006 and 0.008, respectively. B. Schematic diagram showing the portion of the sequence used to monitor inhibition of RNA synthesis by platinum complexes. The arrows indicate the start of the SP6 and T7 RNA polymerase, which used as a template the bottom or upper strand of the pSP73KB DNA, respectively. (●) major stop signals (from Figure **S1**A) for DNA modified by cisplatin; (○) stop signals for DNA modified by ACR. The numbers correspond to the nucleotide numbering in the sequence map of the pSP73KB plasmid. The red frame indicates 5‘-CG sequences where the stop signals of ACR occurred.

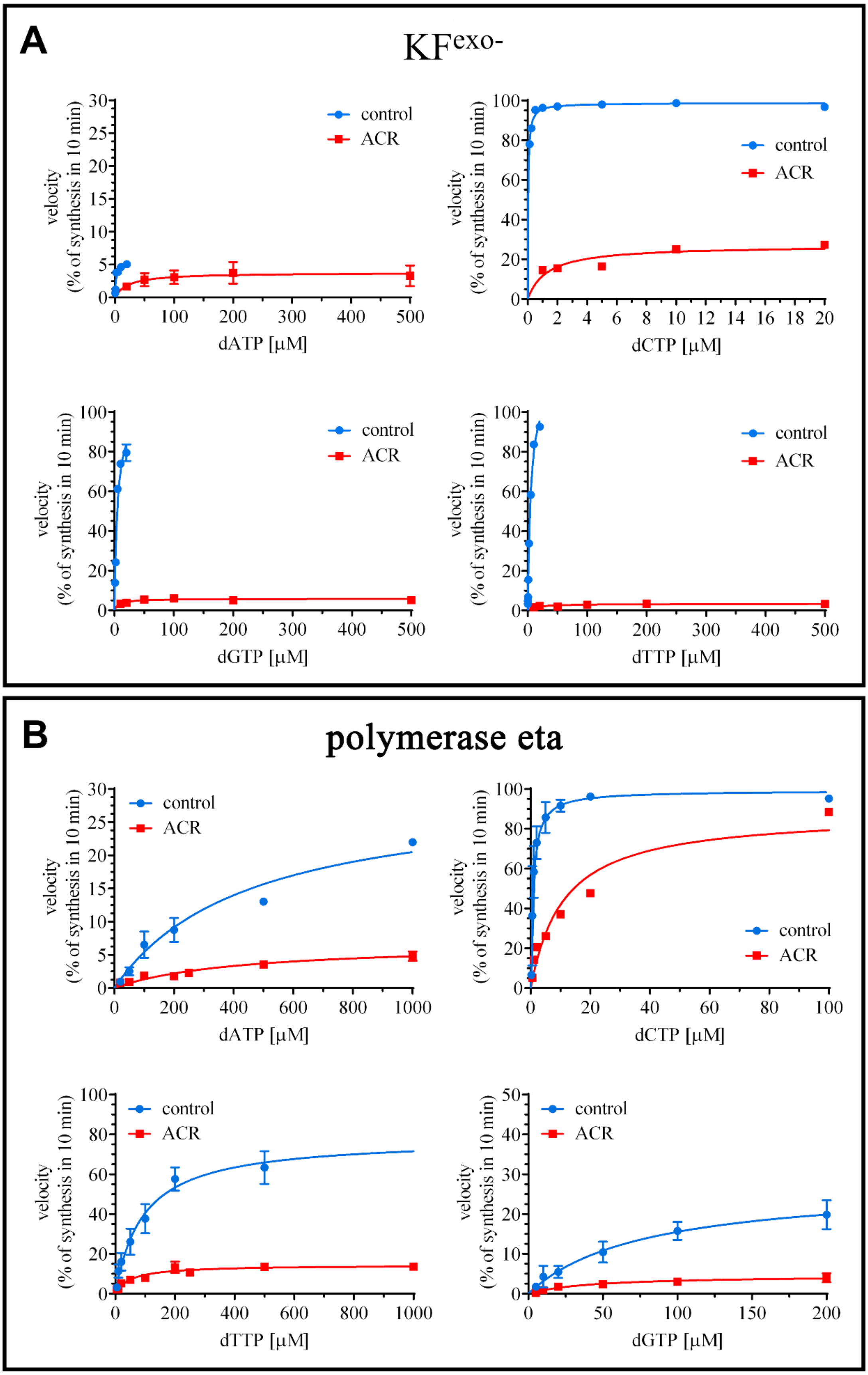

Figure S2. Steady-state kinetic analysis-graphical representation of individual dNTP insertions by KF^{exo-} (panel **A**) and human polη (panel **B**). Data are means (±SEM) from at least two different experiments; for some points, the error bar is shorter than the height of the symbol. Fitting was to a hyperbolic equation in GraphPad Prism v. 7.04, and V_{max}, t, and K_m values are presented in Table S1 and S2, respectively.

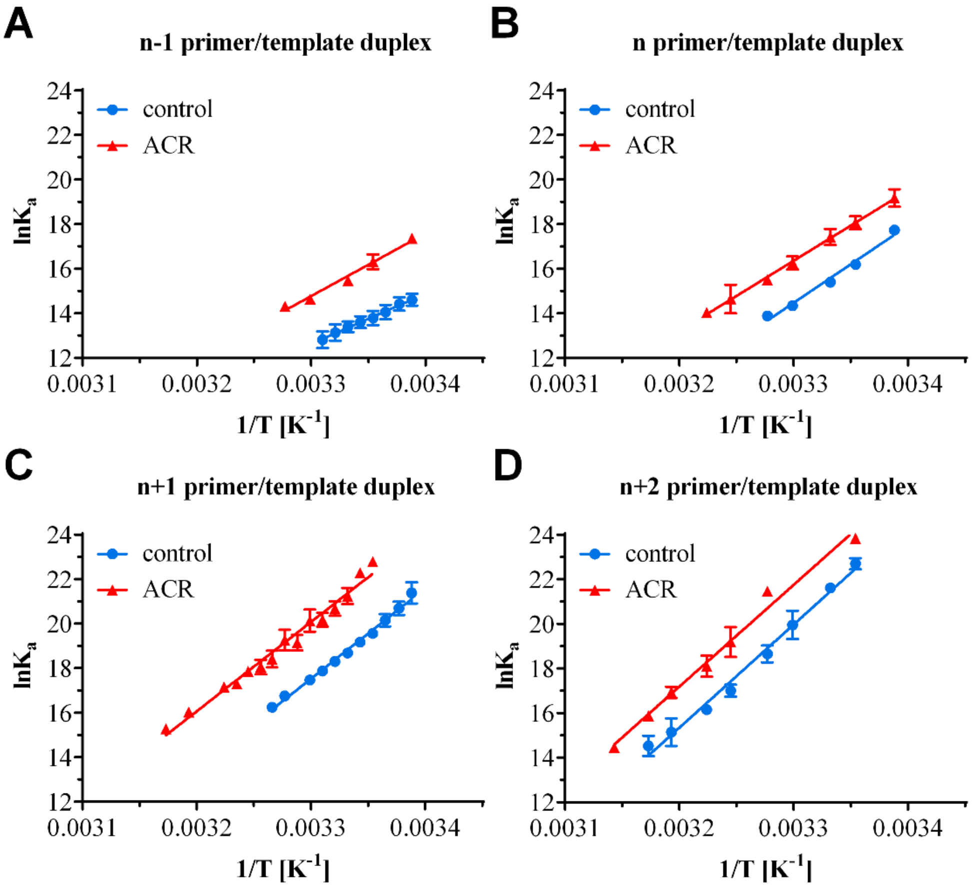

Figure S3. Microscale thermophoresis (MST) determination of the thermodynamic parameters of DNA constructs designed to simulate translesion DNA synthesis (TLS). Van't Hoff plots of the n-1 (A), n (B), n+1 (C) or n+2 (D) primer-template hybridization reactions. K_d values were calculated for each temperature by fitting the T-Jump or thermophoresis signal and plotted as $\ln(K_a = 1/K_d)$ vs. $1/T$ (K). ΔH was obtained from the slope m of the linear fit as $m = -H^o/R$. Under the assumption that ΔH is constant in the relatively small linear range of the van't Hoff plot, ΔS was directly derived from the plot as $y(0) = \Delta S^o/R$. The universal gas constant $R = 8.314$ J $K^{-1}mol^{-1}$. Data are means (±SD) from at least two different experiments, for some points, the error bar is shorter than the height of the symbol; coefficient of determination $r^2 > 0.98$.

Table S1. Steady-state kinetics of the incorporation of dNTP by **KF^{exo-}**. The dNTP incorporation opposite the position of the adduct and extended (17+) in 24-mer DNA template platinated by ACR or unplatinated control, which formed duplex with 16-mer primer (n - 1). n = complementary **C** or non-complementary A, G, or T nucleotide in extended primer.

Control duplex	V_{max} (% min^{-1})	K_m (μM)	V_{max}/K_m	f[a]	RF[b]
**n = C**	9.88 ± 0.06	0.033 ± 0.003	301.0		
n = A	0.578 ± 0.051	2.39 ± 0.65	0.242	0.00080	
n = G	10.5 ± 0.9	5.14 ± 1.23	2.043	0.00679	
n = T	12.2 ± 0.6	5.42 ± 0.71	2.251	0.00748	

Duplex containing ACR	V_{max} (% min^{-1})	K_m (μM)	V_{max}/K_m	f[a]	RF[b]
**n = C**	2.70 ± 0.34	1.33 ± 0.71	2.024		0.01
n = A	0.378 ± 0.088	21.9 ± 16.5	0.017	0.00840	0.07
n = G	0.579 ± 0.061	7.21 ± 5.32	0.080	0.03953	0.04
n = T	0.334 ± 0.049	13.1 ± 9.7	0.025	0.01235	0.01

[a] misincorporation frequency $f = (V_{max}/K_m)_{incorrect\ dNMP}/(V_{max}/K_m)_{correct\ dNMP}$

[b] relative efficiency *RF* compares the efficiency (V_{max}/K_m) of the particular dNTP insertion opposite ACR adduct in 5'-TC**G**T- (24-mer) templates to the efficiency of the same dNTP insertion opposite unmodified **G** in control, unplatinated template.

Table S2. Steady-state kinetics of the incorporation of dNTP by human DNA **polymerase eta**. The dNTP incorporation opposite the position of the adduct and extended (17+) in 24-mer DNA template platinated by ACR or unplatinated control, which formed duplex with 16-mer primer (n - 1). n = complementary **C** or non-complementary A, G or T nucleotide in extended primer (17+).

Control duplex	V_{max} (% min^{-1})	K_m (μM)	V_{max}/K_m	f[a]	RF[b]
**n = C**	9.91 ± 0.64	0.802 ± 0.233	12.37		
n = A	2.97 ± 0.78	450 ± 206	0.007	0.00057	
n = G	2.76 ± 0.64	77.5 ± 40.8	0.036	0.00292	
n = T	7.82 ± 0.85	93.2 ± 27.9	0.084	0.00679	

Duplex containing ACR	V_{max} (% min^{-1})	K_m (μM)	V_{max}/K_m	f[a]	RF[b]
**n = C**	8.80 ± 0.43	11.0 ± 2.6	0.802		0.06
n = A	0.679 ± 0.094	414 ± 128	0.002	0.00249	0.29
n = G	0.464 ± 0.082	42.3 ± 20.6	0.011	0.01372	0.31
n = T	1.44 ± 0.21	51.4 ± 23.1	0.028	0.03491	0.33

The footnotes [a, b] have the same meaning as those under Table S1.

Table S3. MST-derived thermodynamic parameters of dissociation of duplexes formed between the 15-mer DNA templates (unmodified control duplex and template platinated by ACR) and primers n - 1, n, n + 1, or n + 2.

Control duplexes[1]	ΔH (kJ mol^{-1})[2]	ΔS (kJ K^{-1} mol^{-1})[2]	ΔG_0^{310} (kJ mol^{-1})[2]	ΔG_0^{298} (kJ mol^{-1})[2]	$K_d^{[310]}$[3] (nM)	$K_d^{[298]}$[3] (nM)
n-1	187	0.511	28.5	34.6	15846	867
n	295	0.853	30.4	40.7	7585	74.0
n+1	339	0.973	37.2	48.9	543	2.71
n+2	385	1.106	42.0	55.2	84.4	0.213

Duplexes containing ACR adduct	ΔH (kJ mol^{-1})	ΔS (kJ K^{-1} mol^{-1})	ΔG_0^{310} (kJ mol^{-1})	ΔG_0^{298} (kJ mol^{-1})	$K_d^{[310]}$[3] (nM)	$K_d^{[298]}$[3] (nM)
n-1	234 (47)	0.649 (0.138)	32.7 (4.2)	40.5 (5.9)	3109	80.2
n	260 (-35)	0.722 (-0.131)	36.1 (5.7)	44.7 (4.0)	832	14.7
n+1	335 (-4)	0.940 (-0.033)	43.5 (6.3)	54.7 (5.8)	47.2	0.261
n+2	380 (-5)	1.080 (-0.026)	45.0 (3.0)	58.0 (3.3)	26.4	0.069

[1]The nucleotide sequences of the templates and primers are shown in Figures 1 and 5.

[2] The ΔH and ΔS values are averages derived from two independent experiments. The uncertainties of the parameters are as follows: ΔH (±3%), ΔS (±5%), ΔG_0 $^{310,\,298}$ (±2%), $K_d^{[298]}$ or $K_d^{[310]}$ (±20%). "ΔΔ" parameters are in parentheses (these parameters are computed by subtracting the appropriate value measured for the control duplex from the value measured for the same duplex containing the monofunctional adduct of ACR. $\Delta G_0^{310,\,298} = \Delta H - T\Delta S$; T = 310 or 298 K. [3] $K_d^{[310]}$ or $K_d^{[298]}$ denote the dissociation constants for strand dissociation at 310 or 298 K, respectively. K_d was obtained after recalculation from the equation $\Delta G_0^{310,\,298} = -RT^{310,\,298} \times \ln K_d$.
